# Human vs. AI in Conducting Scoping Reviews: Evaluating Large Language Model Accuracy Across Article and Task Type

**DOI:** 10.1093/geroni/igaf122.2181

**Published:** 2025-12-31

**Authors:** Chang Yu, Mo Han, Rui Huang, Hanna Grol-Prokopczyk, Gongda Yu

**Affiliations:** University at Buffalo, SUNY, Getzville, New York, United States; University at Buffalo, Buffalo, New York, United States; University at Buffalo, Buffalo, New York, United States; University at Buffalo, Buffalo, New York, United States; University at Buffalo, Buffalo, New York, United States

## Abstract

**Objectives:**

Large language models (LLMs), such as ChatGPT’s, are increasingly used to assist with health- and aging-related scoping reviews, which are often very time-consuming when done by humans alone. However, empirical evidence on the reliability of LLMs in extracting data from peer-reviewed literature (content coding) remains limited. This study evaluates the accuracy of ChatGPT-4o’s content coding across different article types (systematic reviews, narrative reviews, and meta-analyses) and task types (e.g., single-select vs. multiple-select questions) by comparing its results to human coding from an existing scoping review.

**Methods:**

We selected 26 articles from a previously human-coded scoping review of 398 articles on social disparities (including age-related disparities) in pain. We then used ChatGPT-4o’s Application Programming Interface (API) to extract and code eight characteristics of each article, including article type, independent variable(s), dependent variable(s), mechanism(s), and research findings, based on predefined options. For single-select questions, agreement between human and AI codings was categorized as same or different. For multiple-select questions, additional categories captured partial agreement.

**Results:**

Human-AI agreement was higher for single-select questions (71%) than for multiple-select questions (29%). By article type, agreement was highest for meta-analyses (85% and 45% for single- and multiple-select questions, respectively), and much lower for systematic reviews in single-select questions (38% agreement) or narrative reviews in multiple-select questions (17% agreement).

**Discussion:**

ChatGPT is helpful for straightforward coding tasks in scoping reviews, but its reliability declines with more complex tasks and diverse article types. These findings highlight the need for careful consideration when incorporating LLMs into scoping reviews.

